# New Orleans Dental Association

**Published:** 1873-11

**Authors:** 


### NEW ORLEANS DENTAL ASSOCIATION.

New Orleans, Oct. 10, 1873.

Dr. Taft:

Dear Sir:—On the third day of July, 1873, at a meeting of the New Orleans Dental Assoeiation, the following preamble and resolutions were passed.

Illness and absence of the Recording Secretary, Prof. A. F. McLaw, M. D., D, D. S., prevented him from forwarding you a copy, which duty I now perform

Yours truly,

Jas. S. Knapp,

Member of the N. O. Dental Association, acting in place of Recording Secretary.

"Whereas, Divine Providence in his all-wise dispensation has seen fit to allow the laws of nature to be exercised in removing from life's busy scenes and cares the very estimable gentleman, Cyrus S. Knapp, D. D. S., at his residence, near Jackson, state of Mississippi, long before having attained that full measure of earthly existence which has been allotted to man; and

Whereas, in his demise, society has been deprived of a useful, worthy member, his wife of a loving, devoted husband, his children and brothers of an affectionate father and brother, whilst his numerous friends and relatives have sustained the irreparable loss of a warm and genial friend, the dental profession of one of its highest ornaments, and this association one of its corresponding members; therefore be it

*Resolved*, that we tender to his family and relatives our heartfelt sympathy and condolence for their sad bereavement; and that this preamble and resolutions be spread upon the minutes of this meeting; and that a copy of the same be forwarded to his grief-stricken family; and that copies be likewise furnished to the dental journals for publication.

